# High Capacity and Superior Rate Performances Coexisting in Carbon-Based Sodium-Ion Battery Anode

**DOI:** 10.34133/2019/6930294

**Published:** 2019-06-25

**Authors:** Yuqian Li, Liyuan Zhang, Xiuli Wang, Xinhui Xia, Dong Xie, Changdong Gu, Jiangping Tu

**Affiliations:** ^1^State Key Laboratory of Silicon Materials, Key Laboratory of Advanced Materials and Applications for Batteries of Zhejiang Province, School of Materials Science and Engineering, Zhejiang University, Hangzhou 310027, China; ^2^Guangdong Engineering and Technology Research Center for Advanced Nanomaterials, School of Environment and Civil Engineering, Dongguan University of Technology, Dongguan 523808, China

## Abstract

Amorphous carbon is considered as a prospective and serviceable anode for the storage of sodium. In this contribution, we illuminate the transformation rule of defect/void ratio and the restrictive relation between specific capacity and rate capability. Inspired by this mechanism, ratio of plateau/slope capacity is regulated via temperature-control pyrolysis. Moreover, pore-forming reaction is induced to create defects, open up the isolated voids, and build fast ion channels to further enhance the capacity and rate ability. Numerous fast ion channels, high ion-electron conductivity, and abundant defects lead the designed porous hard carbon/Co_3_O_4_ anode to realize a high specific capacity, prolonged circulation ability, and enhanced capacity at high rates. This research deepens the comprehension of sodium storage behavior and proposes a fabrication approach to achieve high performance carbonaceous anodes for sodium-ion batteries.

## 1. Introduction

Recently, many alternatives of lithium-ion batteries (LIBs), which are restricted by the rising costs, are investigated to satisfy the demand of energy storage. Particularly, sodium-ion batteries (SIBs) are widely researched due to (1) abundant resource (23600 ppm in the earth crust, 1000 times more than lithium) and the even distribution of sodium, (2) low cost of sodium, and (3) similar physicochemical properties to lithium [1–3]. Therefore, the successful experience of cathode for LIBs might be copied to SIBs firsthand [4, 5]. Unfortunately, some decent-performance anodes of LIBs in current research, such as Sn [6], Si [7], and their alloys, titanium-based salts [8], and metal oxides/sulfides/phosphides or their analogues (Fe_3_O_4_ [9], MnO [10], Mn_3_O_4_ [11], Co_3_O_4_ [12, 13], SnO_2_ [14], CoSe_2_ [15], CoS_2_ [16], MoS_2_ [17–19], and Sn_4_P_3_ [20]), are problematic in SIBs. Hence, it is of great significance to develop suitable anodes for SIBs.

Graphite is the promising and competitive anode for LIBs. Nevertheless, it has not exhibited equivalent performance for SIBs, which is probably due to the larger radius of Na^+^ [21, 22]. At present, kinds of other amorphous carbonaceous materials that possess large interlayer distance and disordered structure have been developed as anodes for SIBs. Hard carbon (HC), including heteroatom-doped hard carbon [7, 23, 24] and biomass-derived carbon [25–31], exhibits strong competitiveness. These amorphous carbon materials can insert/extract sodium ions at a low voltage [25, 32–34].

Amorphous carbon exhibits distinct behaviors of sodium storage at different voltages. Thereby, the charge-discharge curve can be divided into slope and plateau segments. There are diverse explanations for the sodium-ion storage behavior of amorphous carbon: insertion-absorption, absorption-insertion, and absorption-filling mechanisms [35]. Among them, absorption-filling mechanism is widely verified [28, 36–38], which means the following: (1) defects, fringes, and the surface among the regional graphitizing area contribute to the slope capacity; (2) nanodomains and nanovoids are ascribed to the reserve of sodium ions in the plateau capacity [39–42].

Inspired by this mechanism, HC can be modified via the following routines: (1) increasing defects and edges to improve the slope capacity, (2) creating more domains and voids to enhance the plateau capacity, and (3) constructing fast sodium-ion channel. Thus, optimal performance can be realized by regulating the ration of slope and plateau region. On one hand, different carbonization temperature can adjust the graphitization degree of carbon, and thereby the ratio of slope region and plateau region is controllable [28, 43, 44]. On the other hand, the pore-forming process can create more voids and build fast sodium-ion channels. Chemical activating reagent (such as KOH [45, 46] and HNO_3_ [45]) and metal chlorides/oxides (including NiCl_2_ [25] and FeO [47]) are adopted to etch substrate of HCs. Subsequently, the substrate loaded numerous defects can be created due to the hollow and loose framework [24, 48, 49]. Cobalt salt ramification (e.g., Co_3_O_4_), significantly, can form more homogenous nanoscale pores and numerous active sites in carbon to produce fast sodium-ion channels [50]. In addition, Co_3_O_4_ is also proposed as a promising potential candidate anodic host material for SIBs [28]. In view of these advantages, temperature-control and pore-forming strategies will open up a novel way to fabricate high-performance carbonaceous anodes for rechargeable SIBs.

In this present work, we illuminate the transformation rules of defect/void ratio which affect the plateau/slope capacity. Moreover, the constraint relationship between specific capacity and rate capability is also explained. Inspired by this correlativity, porous hard carbon/Co_3_O_4_ particles (PHC/Co_3_O_4_) are prepared via the temperature-control carbonization (to achieve the optimal structure) and the pore-forming strategy (to build fast ion channels and ameliorate the retention ratio at high rate). Owing to the optimal defect/void ratio and fast ion channels, the PHC/Co_3_O_4_ anode exhibits excellent sodium-ion insertion/extraction performance with high reversible capacity, prolonged cyclic ability, and enhanced capacity at high rate. Our research proposes a synthetic method for preparation of economic carbonaceous anode for SIBs based on the sodium storage mechanism.

## 2. Results and Discussion

**Figure 1:**
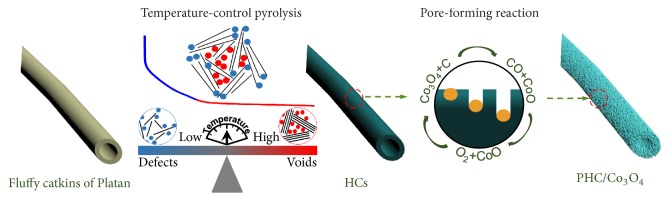
General diagram of the preparation process and formation mechanism of PHC/Co_3_O_4_.

Platan tree plays an important role in building a beautiful urban environment in most cities. The fluffy catkins of platan fruit act as a raw material of HC due to its natural abundance and renewability. Cutting the platan fruit to fructus, plenty of fluffy catkins with diameters of 4 cm closely grown on the hard core (1.85 cm) can be seen ([Supplementary-material supplementary-material-1]). Additionally, the fluffy catkins possess a tubular structure with circular radius of 15-20 *μ*m and the tube wall is 1.5 *μ*m in the enlarged drawing.  Figure 1 illuminates the transformation from fluffy catkins to PHC/Co_3_O_4_. There are two processing steps: temperature-control pyrolysis and pore-forming reaction.

### 2.1. Temperature-Control Pyrolysis

**Figure 2:**
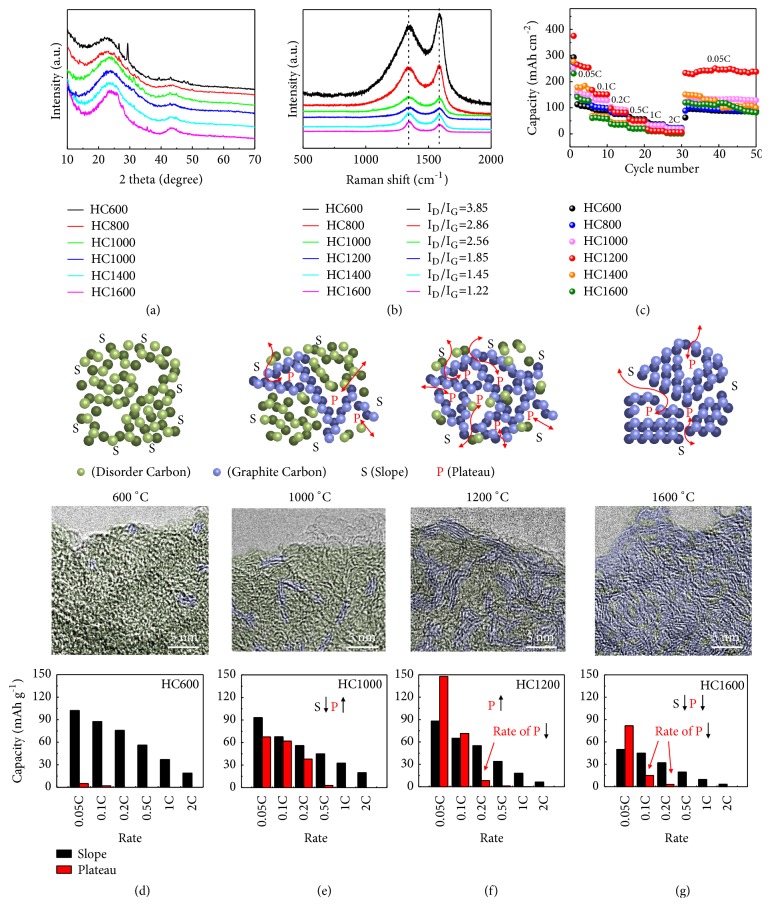
(a) XRD patterns and (b) Raman spectra of HCs. (c) Rate performance of HCs (1 C=300 mA g^−1^). The transformation mechanism of defects/voids and specific slope/plateau capacities of HCs treated at (d) 600°C, (e) 1000°C, (f) 1200°C, and (g) 1600°C.

The ratio of defects and voids in HCs is adjusted by a simple pyrolysis process from 600°C to 1600°C (namely, HC600 to HC1600). With increasing the temperature, the graphitization degree of HCs is enhanced. Graphitization means the reduction of defects and the formation of voids. X-ray diffraction (XRD) and Raman spectra are used to characterize the interior structure of HCs (Figures 2(a) and 2(b)). There are two broad peaks at around 24° and 43° in XRD patterns. These two broad peaks refer to (002) and (101) diffraction arrangements and indicate the dominating amorphous structure in HCs (JCPDS 75-1621). Noticeably, the (002) peaks shift to higher angle slightly from 600°C to 1600°C due to the increase of graphitization as the defect-dominated arrangement. Some impurity peak of HC600 is ascribed to the incomplete pyrolysis. Moreover, the Raman spectra exhibit two characteristic bands. The differences in Raman spectra among HCs are attributed to the transformation of graphitic degree. As shown in Figure 2(b), D band (~1340 cm^−1^) and G band (~1590 cm^−1^) are manifest and the values of* I*_*D*_*/I*_*G*_ are 3.85, 2.86, 2.56, 1.85, 1.45, and 1.22 corresponding to the different pyrogenic temperatures. The decrease of values (*I*_*D*_*/I*_*G*_) indicates the growth of atomic configuration.

Cyclic voltammetry (CV) tests in the first 5 cycles of all HCs are carried out at a specific scanning speed (0.1 mV s^−1^). All the HCs exhibit the distinct oxidation peak in the first cycle due to the formation of solid electrolyte interface (SEI) ([Supplementary-material supplementary-material-1]). HC600 exhibits differentiated curves due to the incomplete carbonization. Beyond that, the other HCs synthesized at different pyrolysis temperature show similar CV curves: all samples exhibit an obvious redox peak at around 0.5 V. To further display the specific capacity change for all HCs treated at different temperature, the CV curves of all HCs in 5th cycle are summarized in [Supplementary-material supplementary-material-1]. It is obvious that the interior area raises from HC600 to HC1200 and decreases to HC1600 subsequently. Consequently, HC1200 shows the largest CV curve area among all HCs, suggesting the largest specific capacity.

Rate and cycling performances of all HCs are evaluated using half-cells (Figures 2(c) and [Supplementary-material supplementary-material-1]). HC1200 shows the highest specific capacity in cyclic performance test. However, the sodium storage behaviors of HC1200 are not perfect, especially the rate capability. To further clarify the capacity change mechanism, the galvanostatic discharge/charge profiles are analyzed at different rates ([Supplementary-material supplementary-material-1]). All the HCs display similar voltage profiles comprising a sloping segment (high voltage) and a plateau segment (low voltage). According to absorption-filling mechanism, the slope and plateau capacities are corresponding to the sodium storage performance of defects and voids, respectively. Brunauer-Emmett-Teller (BET) results are conducted to prove the assumption. [Supplementary-material supplementary-material-1] shows the pore size distribution of HCs (from HC800 to HC1400). The specific surface area and the number of pores decrease with the increase of carbonization temperature, indicating the decrease of defects. This is owing to the increase in graphitic degree and the agglomeration of pores in graphite layers.

The change rule of slope and plateau capacity of all the HCs is presented clearly in Figures 2(d)–2(g). HC600 is full of defects and a few voids (Figure 2(d)), which exhibits a high slope capacity and low plateau capacity. With the increase of pyrolysis temperature, the voids in HC1000 increase significantly and the defects decrease slightly due to the cross-pile-up of graphitization carbon (Figure 2(e)), whereas too high degree of graphitization for HCs will cause orderly-pile-up of graphitization carbon, resulting in significant reduction of voids and ion channels. This process is presented by the high-resolution TEM (HRTEM) images vividly (Figures 2(d)–2(g) and [Supplementary-material supplementary-material-1]). The graphite layers are short and disordered when carbonized at low temperature. The chaotic layers will elongate and arrange in order when the carbonized temperature increases. Variation of capacity is caused by the change of structure. As shown in Figure 2(g), both the slope and plateau capacities of HC1600 decrease. Moreover, the initial Coulombic efficiencies are shown in [Supplementary-material supplementary-material-1]. [Supplementary-material supplementary-material-1] summarizes the variation of plateau/slope capacity. Therefore, it is concluded that (a) the high capacity anode with excellent rate performance due to the interinhibitive relationship between defects and voids is hard to acquire and (b) optimal pyrolysis temperature will help in achieving the high volume of voids and optimizing the ratio of slope and plateau capacities. Taking all these factors into consideration, HCs cannot be acquired simultaneously without further modification.

### 2.2. Pore-Forming Reaction

HC1200, the highest capacity sample, shows a poor rate performance (Figure 2(f)). Inspired by the mechanism discussed above, the pore-forming strategy can open the fast transfer channels for sodium ions and break the bottleneck of capacity. The pore-forming process is also illustrated in Figure 1. HCs are immersed in Co(CH_3_COO)_2_ solution and heated at 400°C in air to form a porous structure. The surface of HCs is confronted with a corrosion process [51]. The specific corrosion process is illustrated as follows:

(1) $$\begin{array}{c} Co{\left(\;{CH}_{3}COO\;\right)}_{2}+2O_{2}+C\overset{heat}{\to }\\ \frac{1}{3}{Co}_{3}O_{4}+\frac{4}{3}CO+\frac{2}{3}{CO}_{2}+C_{2}H_{4} \end{array}$$

(2) $$\begin{array}{c} \frac{1}{3}{Co}_{3}O_{4}+\frac{1}{3}C\overset{heat}{\to }\\ \frac{1}{3}CO+CoO\\ 2{CO}_{2}+2H_{2}O \end{array}$$

(3) $$\begin{array}{c} CoO+\frac{1}{6}O_{2}\overset{heat}{\to }\frac{1}{3}{Co}_{3}O_{4}\\ 2{CO}_{2}+2H_{2}O \end{array}$$

(4) $$\begin{array}{c} \frac{4}{3}CO+\frac{2}{3}O_{2}\overset{heat}{\to }\frac{4}{3}{CO}_{2}\\ 2{CO}_{2}+2H_{2}O \end{array}$$

(5) $$\begin{array}{c} C_{2}H_{4}+3O_{2}\overset{heat}{\to }\\ 2{CO}_{2}+2H_{2}O \end{array}$$

Carbon reacts with Co(CH_3_COO)_2_ and results in the formation of Co_3_O_4_ (see (1)). In this process, Co_3_O_4_ is the key material to form porous structure. Mutual conversion of Co_3_O_4_ and CoO consumes part of the carbon. During the heat treatment process, the chain reactions (see (2) and (3)) proceed continuously and porous carbon with abundant defects is prepared.

**Figure 3:**
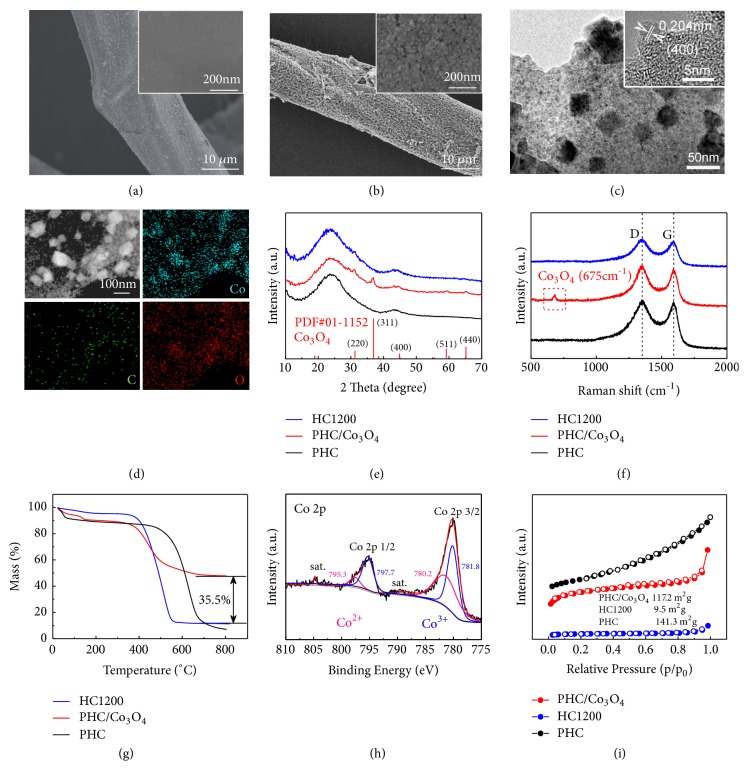
Characterization results of HC1200, PHC/Co_3_O_4_, and PHC. SEM images of (a) HC1200 and (b) PHC/Co_3_O_4_; (c) TEM images PHC/Co_3_O_4_; (d) EDS elemental mapping of PHC/Co_3_O_4_; (e) XRD patterns, (f) Raman spectra and (g) TG results of HC1200, PHC/Co_3_O_4_, and PHC; (h) XPS Co 2p spectrum of PHC/Co_3_O_4_. (i) Nitrogen adsorption/desorption isotherms of HC1200 and PHC/Co_3_O_4._

The SEM image of PHC/Co_3_O_4_ displays a rough surface, while HC1200 demonstrates a relatively smooth surface (Figures 3(a)-3(b)). The high-magnification SEM images (Figure 3(b) inset and [Supplementary-material supplementary-material-1]) present that the close-packed sags and crests are formed on the surface of tubular HCs after the reactions. Moreover, HRTEM images can display the graphitic layer. There are many disordered regions in PHC/Co_3_O_4_, and these regions are composed of curved parallel graphene layers (Figure 3(c)). [Supplementary-material supplementary-material-1] displays carbon edges on the surface, which serve as important active sites for insertion and deintercalation of sodium ions. In addition, a HRTEM micrograph of porous carbon tube is shown in [Supplementary-material supplementary-material-1]. The distances between two layers are 0.246 nm and 0.204 nm referring to the (311) and (400) crystal faces in Co_3_O_4_, respectively. Figures [Supplementary-material supplementary-material-1]-[Supplementary-material supplementary-material-1] show the TEM images of PHC. Compared with HC1200, the overall surface of PHC changes from “flatter” to “sags and crests”. Besides, the energy dispersive spectrometer (EDS) is conducted to reveal the distribution of Co_3_O_4_ in PHC/Co_3_O_4_ (Figure 3(d)). The undertint outline refers to carbon substrate and white particles refer to Co_3_O_4_. C, Co, and O elements match well with the image, which offers new evidence for the homogenous distribution of Co_3_O_4_.

The microstructures of HC1200, PHC/Co_3_O_4_, and PHC are also analyzed by XRD and Raman spectroscopy. The XRD patterns illustrate the interior structure and ingredient (Figure 3(e)). Two broaden diffraction peaks located at 24° and 43° in the XRD patterns suggest the similar disordered and amorphous structure of three samples. Moreover, the distinct characteristic peaks of Co_3_O_4_ phase (JCPDS 01-1152) can be seen in the pattern of PHC/Co_3_O_4_. As shown in Figure 3(f), there are two broadened peaks in the same position with the same* I*_*D*_*/I*_*G*_ value (1.85) for all samples in Raman spectra and an extra typical cobalt oxide peak at 675 cm^−1^ appears only in PHC/Co_3_O_4_. Thermogravimetric analysis (TGA) is conducted for testing the weight change of PHC/Co_3_O_4_ and HC1200 in air. Comparative thermograms of three samples are shown in Figure 3(g). The profile of PHC/Co_3_O_4_ has a higher residue after heating to 800°C revealing the Co_3_O_4_ content. A mass loss at low temperature (lower than 180°C) in both PHC/Co_3_O_4_ and PHC is attributed to the higher moisture. The content of Co_3_O_4_ is measured by inductively coupled plasma-optical emission spectrometry (ICP). As shown in [Supplementary-material supplementary-material-1] and [Supplementary-material supplementary-material-1], the content of Co_3_O_4_ is 18.11 wt.% in PHC/Co_3_O_4_. Correspondingly, the measured Co_3_O_4_ content in PHC is 0.01 wt.% which is negligible. From the XPS full spectra, obvious Co peaks only appear in PHC/Co_3_O_4_ ([Supplementary-material supplementary-material-1]). The Co 2p spectrum contains two oblique Co2p_1/2_ (795.3 eV and 797.7 eV) and Co2p_3/2_ (780.2 eV and 781.8 eV) peaks revealing the presence of Co^2+^ and C_O_^3+^ (Figure 3(h)), corresponding to the cobalt oxide CoO and Co_2_O_3_ in Co_3_O_4_ [52]. To characterize the specific surface area of PHC/Co_3_O_4_, nitrogen adsorption/desorption isotherms are performed. As we expected, PHC/Co_3_O_4_ shows supernal pores and higher specific surface area (117.2 m^2^ g^−1^) than HC1200 (9.5 m^2^ g^−1^) (Figure 3(i)). In addition, PHC possesses a higher surface area than PHC/Co_3_O_4_ due to wiping off from Co_3_O_4_.

**Figure 4:**
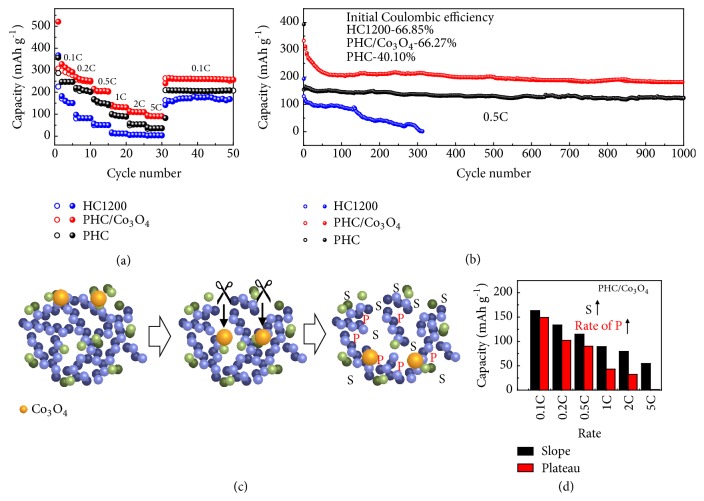
Electrochemical performance of the half-cells and the mechanism. (a) Rate performance and (b) cycling performance of HC1200, PHC/Co_3_O_4_, and PHC anodes. (c) The action mechanism of Co_3_O_4_ as catalyst to increase the slope and plateau capacity. (d) Slope and plateau capacities of PHC/C at different rates.

The sodium-ion storage properties of PHC/Co_3_O_4_, PHC, and HC1200 electrodes are evaluated via half-cells with 1 M NaClO_4_ electrolyte. The CV curve of PHC/Co_3_O_4_ shows a little broader area, and the redox peak at 0.54 V refers to the overlap of Co_3_O_4_ and carbon redox peaks ([Supplementary-material supplementary-material-1]). The CV curve at a low scan rate of 0.05 mV s^−1^ is shown in [Supplementary-material supplementary-material-1]. The redox peaks of Co_3_O_4_ at about 1.6 V, 0.36 V, and 0.96 V can be identified [12, 53]. When Co_3_O_4_ is removed by HNO_3_, the CV curve of PHC returns to the spiculate redox peak ([Supplementary-material supplementary-material-1]).  Figure 4(a) represents the rate performances of PHC/Co_3_O_4_, PHC, and HC1200 electrodes between 0.1 C and 5 C. Evidently, the PHC/Co_3_O_4_ electrode shows the highest capacity. The capacity of PHC/Co_3_O_4_ is about 100 mAh g^−1^ at 5 C, while HC1200 is less than 5 mAh g^−1^. The highest capacity of PHC/Co_3_O_4_ is contributed to the synergistic of Co_3_O_4_ and rich-defect carbon. In addition, the initial Coulombic efficiency of PHC/Co_3_O_4_ (66.27%) is similar to HC1200.  Figure 4(b) exhibits the long cycling results of three samples. The PHC/Co_3_O_4_ electrode preserves a high capacity (200 mAh g^−1^) that can remain stable (remain 87%) after 1000 cycles, superior to PHC (148 mAh g^−1^) and HC1200 (serious capacity deterioration). Moreover, Co_3_O_4_ in PHC/Co_3_O_4_ (18.114 wt.%) can provide certain extra capacity and the synergistic effect of Co_3_O_4_ and PHC is also contributed to the enhanced capacity (details are shown in computation part in Supporting Information). In addition, the Coulombic efficiency of PHC/Co_3_O_4_ is over 99% during cycling (except for the first cycle). As a reference, pure commercial Co_3_O_4_ displays a serious capacity deterioration to about 5 mAh g^−1^ after only 4 cycles ([Supplementary-material supplementary-material-1]). Advantages of the designed porous structure of PHC/Co_3_O_4_ with enhanced electrochemical performance are emphasized here: (1) shorten the ion/electron transfer path which is favorable for high rate applications, (2) amplify the full-cell voltage because HC possesses a low and explanate redox potential, (3) accelerate the shuttle of electrolyte in the HC anode, and (4) enhance the slope capacity resulting from the presence of defects and edges.

The diagrams of the galvanostatic discharge/charge profiles at different rates are further analyzed to prove this hypothesis ([Supplementary-material supplementary-material-1]).  Figure 4(c) describes the action mechanism of Co_3_O_4_ to increase the slope and plateau capacity. HC1200 presents the highest capacity due to the optimal plateau/slope ratio. The pore-forming reaction creates numerous defects (slope capacity), builds fast ion channels for the nanovoids (plateau capacity), and provides additional capacity by residual Co_3_O_4_.  Figure 4(d) shows a clear capacity distribution in the slope and plateau capacities of PHC/Co_3_O_4_. Compared with HCs, the retentions of both slope and plateau capacities of PHC/Co_3_O_4_ at high rates are greatly enhanced via pore-forming strategy.

**Figure 5:**
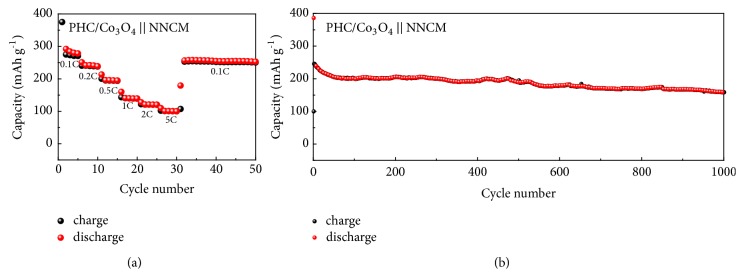
Electrochemical performance of PHC/Co_3_O_4_/NNCM full batteries. (a) Rate performance and (b) long cycling performance.

To further demonstrate its potential applications for SIBs, the PHC/Co_3_O_4_ anode is coupled with Na(Ni_0.8_Co_0.1_Mn_0.1_)O_2_ (NNCM) cathode to assemble full cells.  Figure 5(a) illustrates that the capacity of PHC/Co_3_O_4_//NNCM is over 100 mAh g^−1^ at a high rate of 5 C and can return back to almost the original capacity of around 270 mAh g^−1^ at a low rate of 0.1 C. Moreover, the charge/discharge curve of full cells is displayed in [Supplementary-material supplementary-material-1]. In addition, the PHC/Co_3_O_4_/NNCM cell delivers a stable and prolonged cycling performance as the specific capacity still retains about 83% after 1000 cycles (Figure 5(b)).

## 3. Conclusions and Outlook

In summary, we have illuminated the mechanism for high-performance PHC/Co_3_O_4_ anode via two processing steps: temperature-control pyrolysis and subsequent pore-forming reaction. Firstly, a relative high-performance hard carbon is successfully prepared by temperature-control pyrolysis. Then, PHC/Co_3_O_4_ is fabricated from the hard carbon via subsequent pore-forming reaction. The pore-forming reaction can create abundant defects and build fast sodium-ion channels to further greatly enhance both capacity and rate capability. Moreover, after the pore-forming process, the residual Co_3_O_4_ offers extra capacity due to the synergistic effect of PHC and Co_3_O_4_. Therefore, the PHC/Co_3_O_4_ anode exhibits a high capacity (270 mAh g^−1^), enhanced capabilities at high rates, and prolonged cyclic stability. These consequences take into account the sodium-ion storage behavior from a new perspective and furthermore deepen the understanding of plateau/slope controllable carbonaceous anode for SIBs.

## Supplementary Materials

Supplementary Materials Experimental Section: computational process of the specific capacity of Co_3_O_4_. Figure S1: (a) planetree fruit; (b) silk stripping from the threadlet; (c) cross section of planetree fruit; (d) optical enlargement of TCF. Figure S2: CV curves of HCs synthesized at (a) 600°C, (b) 800°C, (c) 1000°C, (d) 1200°C, (e) 1400°C, and (f) 1600°C at the first 5 cycles. Figure S3: CV curve of HCs between 0.01 and 2.5 V at a scan rate of 0.1 mV s^−1^ at 5th cycle. Figure S4: cycling performance of HCs at 0.1C. Figure S5: charging and discharging curves of HCs synthesized at (a) 600°C, (b) 800°C, (c) 1000°C, (d) 1200°C, (e) 1400°C, and (f) 1600°C at different rate. Figure S6: (a) micropore and (b) mesopore size distribution of HCs (HC800 to HC1400). Figure S7: HRTEM images of (a) HC600, (b) HC1000, (c) HC1200, and (d) HC1600. Figure S8: initial Coulombic efficiencies of HCs. Figure S9: capacity and plateau/slope ratio of all HCs. Figure S10: (a, b) TEM images of PHC/Co_3_O_4_; (c-d) TEM images of PHC. Table 1: the ICP signals of 200 ml HNO_3_ solution (enough to dissolution Co_3_O_4_) immersed in 15 mg PHC/Co_3_O_4_  and PHC then taking out 20 ml filtered solution attenuation to 200 ml and the standard samples. Figure S11: the Co ion intensity signal images of PHC/Co_3_O_4_, PHC, and standard sample attenuation solutions tested by ICP. Figure S12: XPS full spectra of HC1200, PHC/Co_3_O_4_, and PHC. Figure S13: CV curves of (a) PHC/Co_3_O_4_ and (b) PHC; (c) PHC/Co_3_O_4_ at a low scan rate of 0.05 mV s^−1^. Figure S14: rate and cycling performances of pure Co_3_O_4_. Figure S15: charging and discharging curves of PHC/Co_3_O_4_ (a) and PHC (b) at different rate. Figure S16: the charge/discharge curve of full cells.
