# Supplementary material for: High Capacity and Superior Rate Performances Coexisting in Carbon-Based Sodium-Ion Battery Anode
Source: Research (Wash D C). 2019 Jun 25;2019:6930294. doi: 10.34133/2019/6930294 (PMC6753608; doi:10.34133/2019/6930294)
Supplement: Supplementary Materials — Experimental Section: computational process of the specific capacity of Co3O4. Figure S1: (a) planetree fruit; (b) silk stripping from the threadlet; (c) cross section of planetree fruit; (d) optical enlargement of TCF. Figure S2: CV curves of HCs synthesized at (a) 600°C, (b) 800°C, (c) 1000°C, (d) 1200°C, (e) 1400°C, and (f) 1600°C at the first 5 cycles. Figure S3: CV curve of HCs between 0.01 and 2.5 V at a scan rate of 0.1 mV s−1 at 5th cycle. Figure S4: cycling performance of HCs at 0.1C. Figure S5: charging and discharging curves of HCs synthesized at (a) 600°C, (b) 800°C, (c) 1000°C, (d) 1200°C, (e) 1400°C, and (f) 1600°C at different rate. Figure S6: (a) micropore and (b) mesopore size distribution of HCs (HC800 to HC1400). Figure S7: HRTEM images of (a) HC600, (b) HC1000, (c) HC1200, and (d) HC1600. Figure S8: initial Coulombic efficiencies of HCs. Figure S9: capacity and plateau/slope ratio of all HCs. Figure S10: (a, b) TEM images of PHC/Co3O4; (c-d) TEM images of PHC. Table 1: the ICP signals of 200 ml HNO3 solution (enough to dissolution Co3O4) immersed in 15 mg PHC/Co3O4 and PHC then taking out 20 ml filtered solution attenuation to 200 ml and the standard samples. Figure S11: the Co ion intensity signal images of PHC/Co3O4, PHC, and standard sample attenuation solutions tested by ICP. Figure S12: XPS full spectra of HC1200, PHC/Co3O4, and PHC. Figure S13: CV curves of (a) PHC/Co3O4 and (b) PHC; (c) PHC/Co3O4 at a low scan rate of 0.05 mV s−1. Figure S14: rate and cycling performances of pure Co3O4. Figure S15: charging and discharging curves of PHC/Co3O4 (a) and PHC (b) at different rate. Figure S16: the charge/discharge curve of full cells. [file 6930294.f1.docx]

Supporting Information

High Capacity and Superior Rate Performances Coexisting Carbon-Based Sodium-Ion Battery Anode

**Yuqian Li^1^, Liyuan Zhang^1^, Xiuli Wang^1,^*, Xinhui Xia^1,^*, Dong Xie^2^, Changdong Gu^1^ and Jiangping Tu^1,^***

*^1^State Key Laboratory of Silicon Materials, Key Laboratory of Advanced Materials and Applications for Batteries of Zhejiang Province, School of Materials Science and Engineering Zhejiang University, Hangzhou 310027, China.*

*^2^Guangdong Engineering and Technology Research Center for Advanced Nanomaterials, School of Environment and Civil Engineering, Dongguan University of Technology, Dongguan 523808, China.*

*Correspondence should be addressed to Xiuli Wang; wangxl@zju.edu.cn, Xinhui Xia; helloxxh@zju.edu.cn and Jiangping Tu; tujp@zju.edu.cn

**Experimental section**

All reagents were of analytical grade and used without further purification.

**Preparation of hard carbons (HCs):** The biomass-derived HCs were prepared by pyrolysis carpo of planetree directly. The fructus of Platan were collected in our campus (Platan is one of the most popular border trees in China) and then cut the hard, pyknotic core inside, washed with deionized water and dried at 60 °C overnight in an oven. The obtained raw material was pyrolyzed at 600 °C to 1400 °C for 2 h under Ar atmosphere in tubular furnace.

**Preparation of porous hard carbon/Co_3_O_4_ particles (PHC/Co_3_O_4_):** Cobalt acetate tetrahydrate (Co(CH_3_COO)_2_·4H_2_O, 98%, Aladdin) was used as the pore-forming agent. For the formation of PHC, pyrolyzed HC obtained was immersed in 5 mmol L^−1^ cobalt acetate tetrahydrate solution, after sonicated for 40 min, the HC particles were filtered from the solution and dried overnight. Then, the HC contained pore producer was heated in air at 400 °C for 1 h and cooling to room temperature to get PHC/Co_3_O_4_ finally. PHC can be prepared by the PHC/Co_3_O_4_ immersing in 0.5 mol L^−1^ HNO_3_ solution to remove the Co element.

**Preparation of cathode:** Na(Ni_0.8_Co_0.1_Mn_0.1_)O_2_ (NNCM) was prepared by the “mixed hydroxide” method according to a previously reported method.[1] Ni(NO_3_)_2_, Co(NO_3_)_2_ and Mn(NO_3_)_2_ with stoichiometric ratio are mixed under N_2_ atmosphere. NH_3_·H_2_O solution is drop into this mixed solution dropwise to form precipitate precursor. Precursor is dried at 60 ^o^C and mixed with Na_2_CO_3_ particles and heated to 900 ^o^C in air atmosphere to form NNCM.

**Characterization of materials:** The microstructures and morphologies of all samples were characterized by Rigaku D/max 2550PC (Cu Kα), Raman spectroscope (Renishaw Raman microscope under 532 nm laser excitation), field-emission scanning (SEM, Hitachi S-4700) and transmission electron microscopy (TEM, FEI Tecnai G2 F20 at 200 kV). Specific surface area and pore diameter distribution were tested by using a Porosity Instrument. Thermogravimetric analysis (TGA) measurements were employed on a Netzsch STA 449C thermal analyzer tested from room temperature to 800 °C in a N_2_ atmosphere. The contents of Co_3_O_4_ were probed by inductively coupled plasma-optical emission spectrometry (ICP-OES, Agilent 725, Agilent Technologies).

**Electrochemical measurement:** The electrochemical measurements of half cells were performed by CR2025 coin-type cells with PHC/Co_3_O_4_ as working electrode, sodium foil as counter electrode, and Whatman glass microfiber (GF/F 1825-025) as the separator. All the electrolyte was 1 M NaClO_4_ dissolved in ethylene/dimethyl carbonate (DMC) (1: 1 in volume). Superabundant amount of Na metal is used as the counter electrode in the half cells, which has areal capacity with a heavy excess than that of the HC. The full cells possess the same structure except the cathode. The HC Cathode and PHC/Co_3_O_4_ anode were prepared by mixing active material, super P and PVDF with a ratio of 8: 1: 1 onto Al foil, and then dried at 80 °C in vacuum overnight. The anode was excessive slightly according to the theoretical capacity and the mass loading of electrodes were about 2 mg cm^−2^. The coin cells were assembled in the Argon-filled glove box. Cyclic voltammetry (CV) tests were carried out on a CHI660C electrochemistry workstation at a scan rate of 0.1 mV s^−1^ in a range from 0.01 to 2.5 V. The galvanostatic charge/discharge tests were recorded on a LAND battery test system between 0.01 and 2.5 V vs Na/Na^+^ at room temperature (25 °C).

**Computational process of the specific capacity of Co_3_O_4_.**

In PHC/Co_3_O_4_, the content of Co_3_O_4_ is 18.114 wt%. The proportion of carbon is:

100%-18.114% = 81.886%

1 g PHC/Co_3_O_4_ is composed of 0.81886 g carbon and 18.114 g Co_3_O_4_.

In PHC, the capacity of 1 g carbon is 148 mAh.

Supposed the specific capacity of carbon in PHC and PHC/Co_3_O_4_ is same, the capacity of carbon in 1 g PHC/Co_3_O_4_ is:

146*0.81886 = 119.554 mAh

The capacity of Co_3_O_4_ in 1 g PHC/Co_3_O_4_ is:

200-119.554 = 80.446 mAh

The specific capacity of Co_3_O_4_ in PHC/Co_3_O_4_ is:

80.446/18.114% = 444.110 mAh g^-1^.

**Reference**

[1] J. Paulsen, J. Dahn, "Studies of the layered manganese bronzes, Na_2/3_[Mn_1-x_M_x_]O_2_ with M= Co, Ni, Li, and Li_2/3_[Mn_1-x_M_x_]O_2_ prepared by ion-exchange," *Solid State Ionics*, vol. 126, no. 1-2, pp. 3-24, 1999.

**
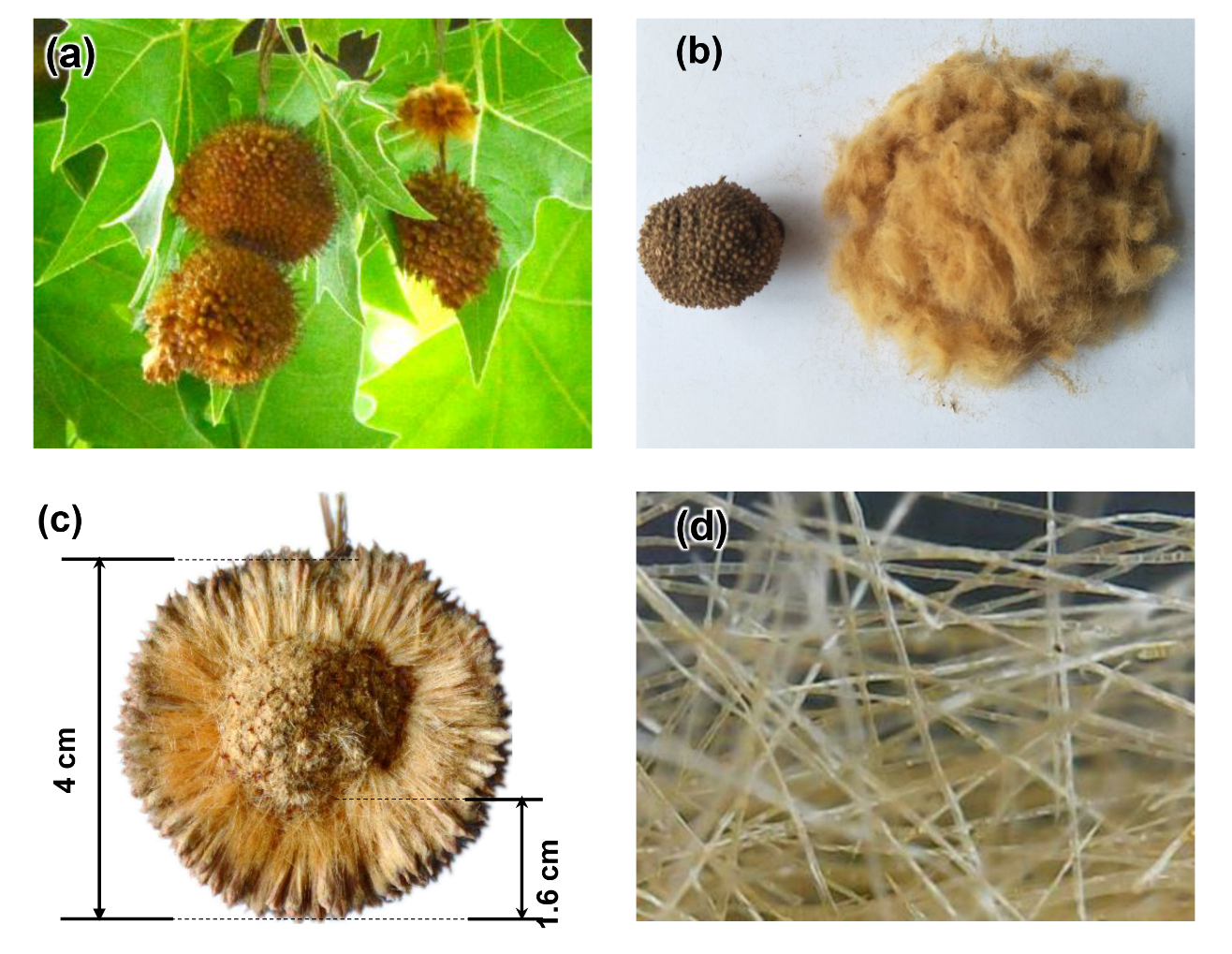
Figure S1** (a) Planetree-fruit of planetree; (b) Silk stripping from the threadlet; (c) Cross section of planetree-fruit; (d) optical enlargement of TCF.

**
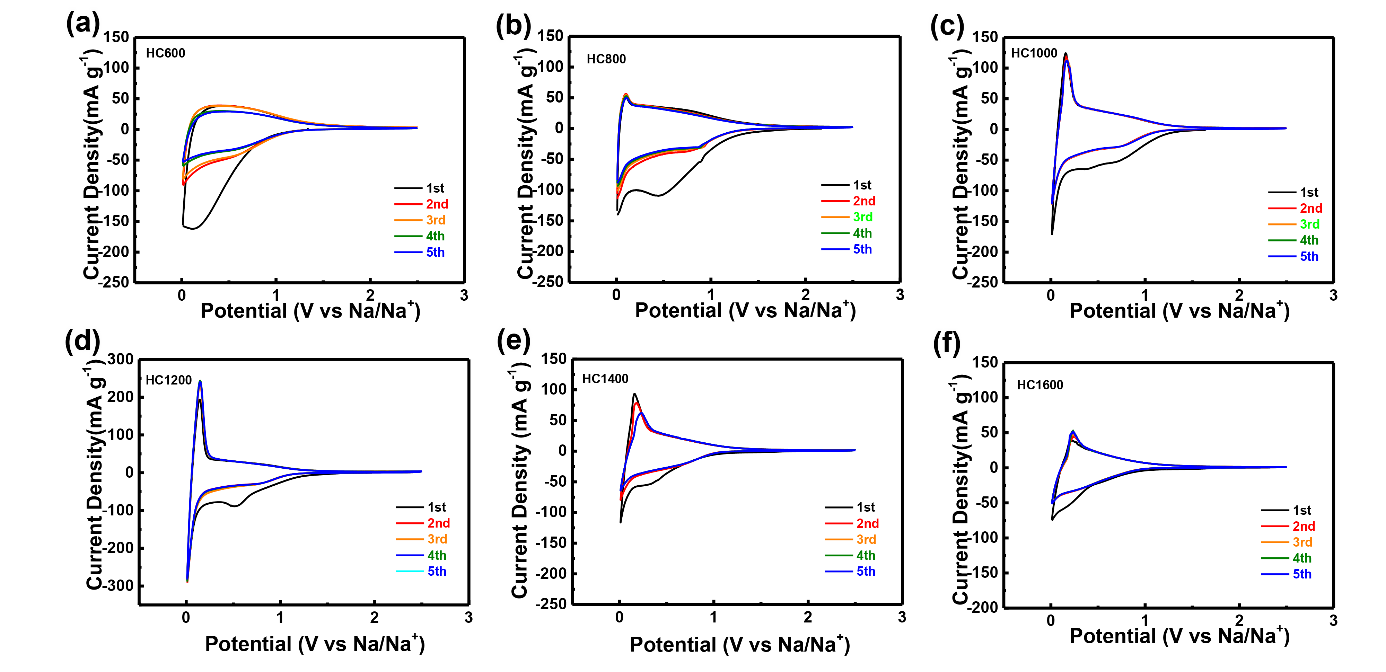
**

**Figure S2** CV curves of HCs synthesized at (a) 600^o^C, (b) 800^o^C, (c) 1000^o^C, (d) 1200^o^C, (e) 1400^o^C, (f) 1600^o^C at the first 5 cycles.

**
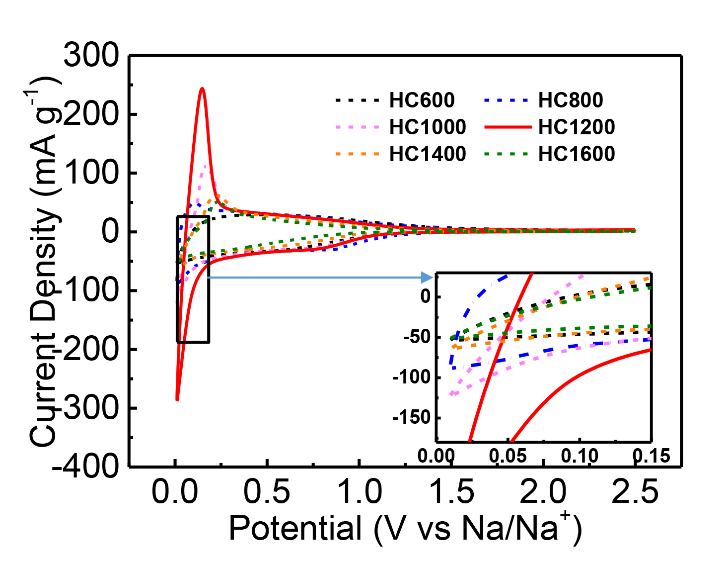
**

**Figure S3** CV curve of HCs between 0.01 and 2.5 V at a scanning rate of 0.1 mV s^−1^ at 5th cycle.

**
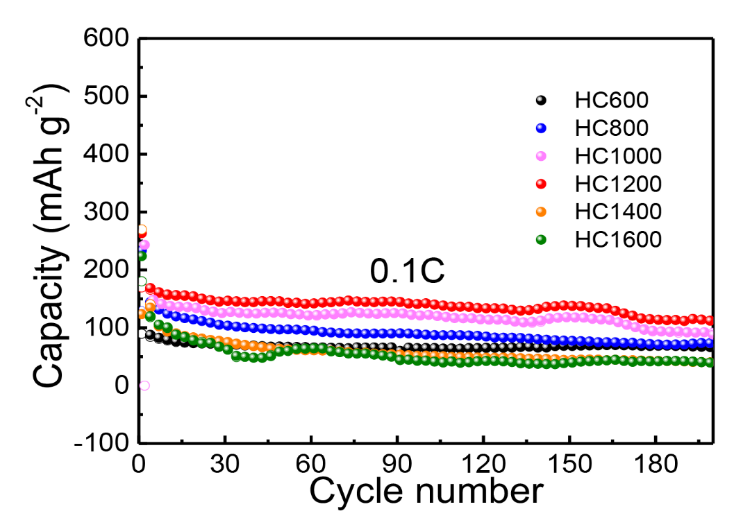
**

**Figure S4** Cycling performance of HCs at 0.1C.

**Figure S5** Charging and discharging curves of HCs synthesized at (a) 600^o^C, (b) 800^o^C, (c) 1000^o^C, (d) 1200^o^C, (e) 1400^o^C, (f) 1600^o^C at different rate.

**
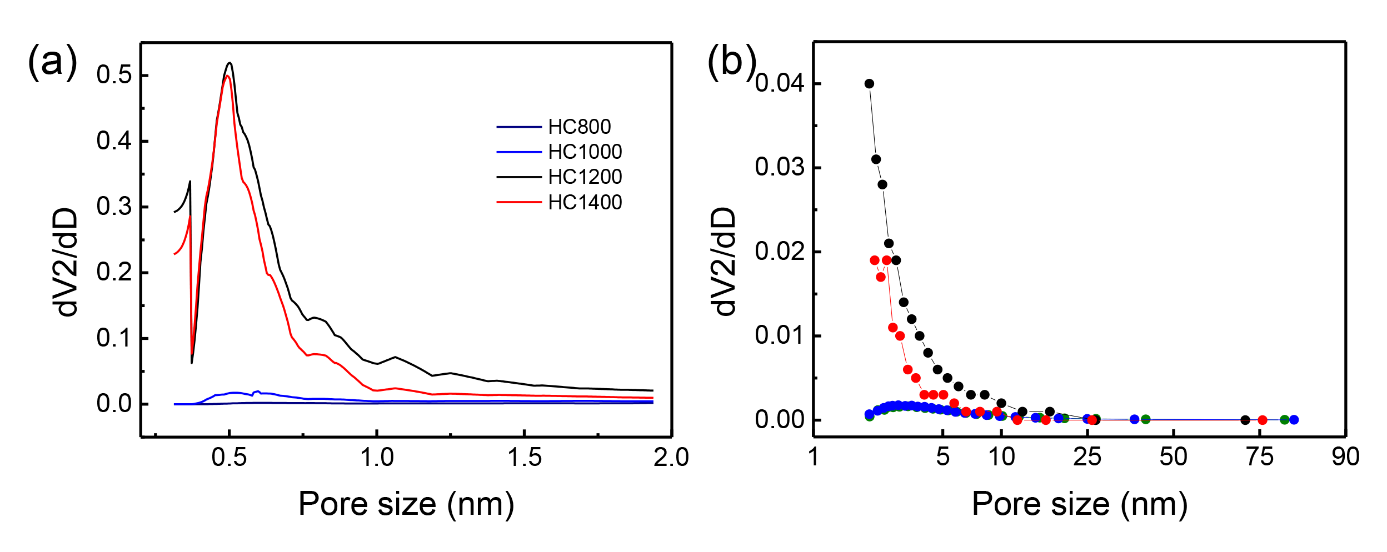
 Figure S6** (a) Micropore and (b) mesopore size distribution of HCs (HC800- HC1400).

**
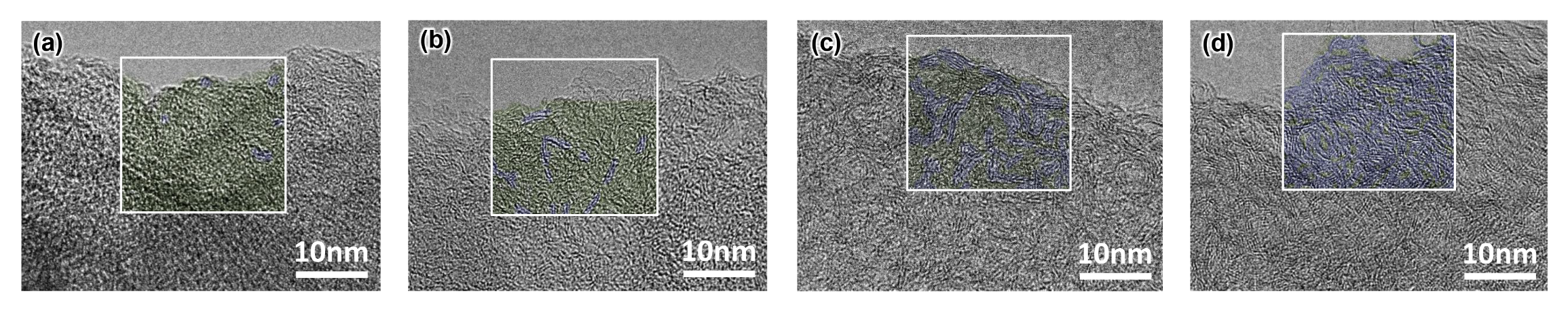

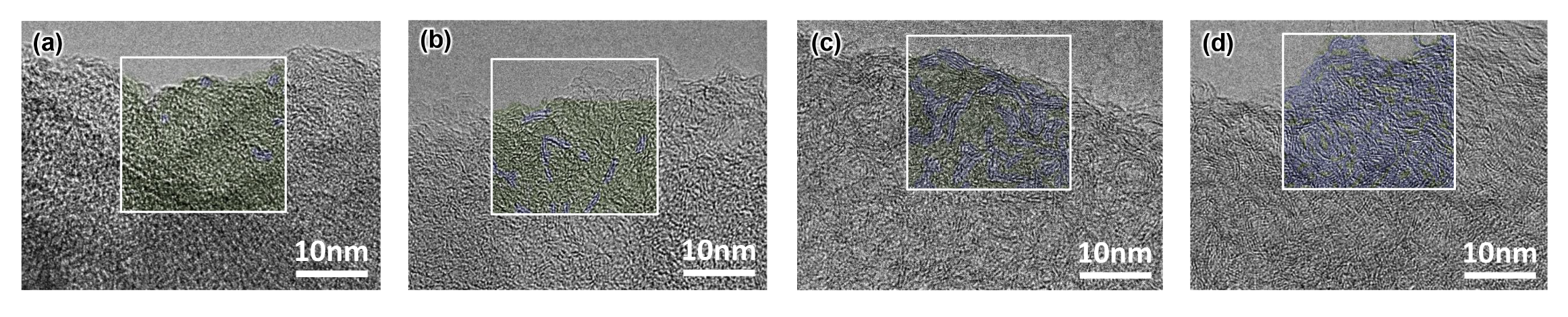
Figure S7** HRTEM images of (a) HC600, (b) HC1000, (c) HC1200 and (d) HC1600.

**
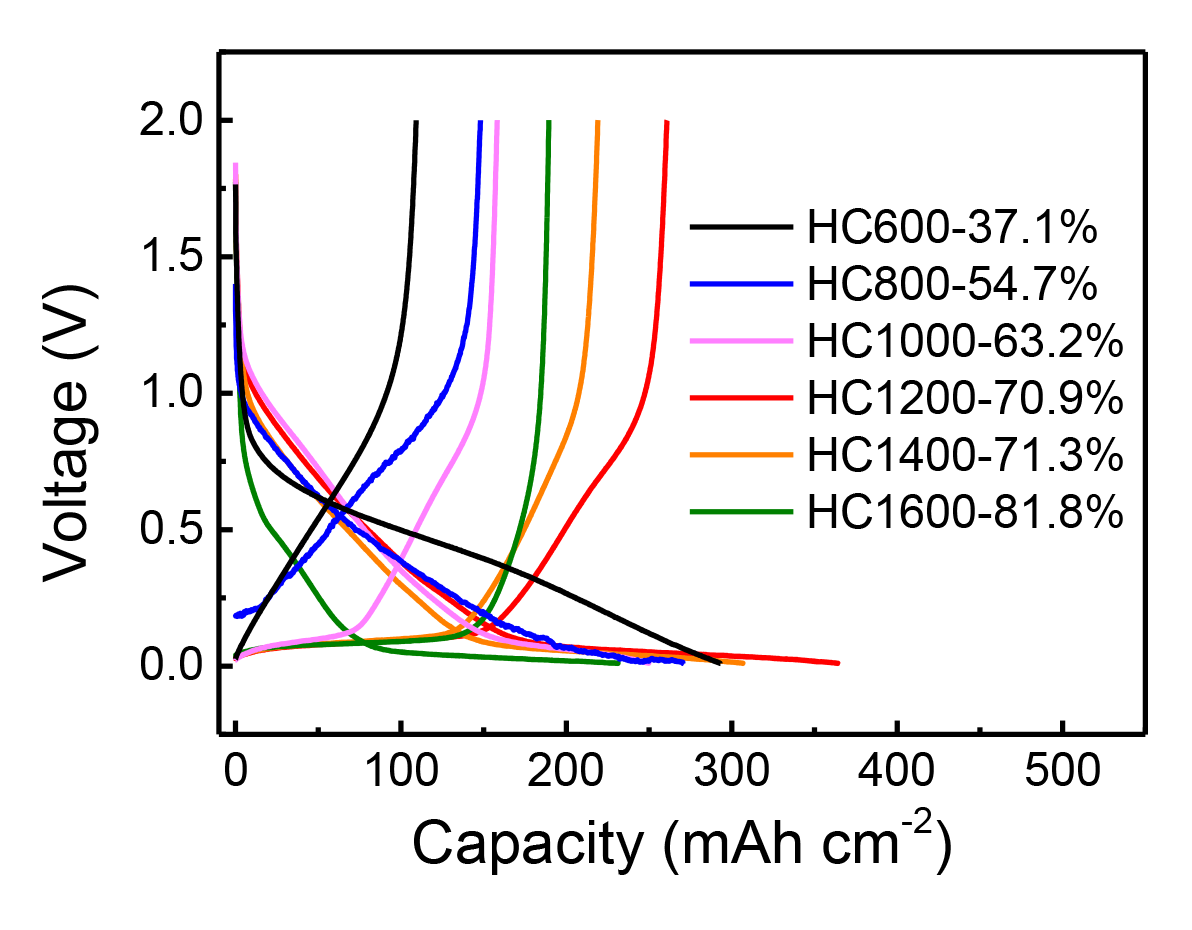
**

**Figure S8** Initial Coulomb efficiencies of HCs.

**Figure S9** Capacity and plateau/slope ratio of all HCs.

**
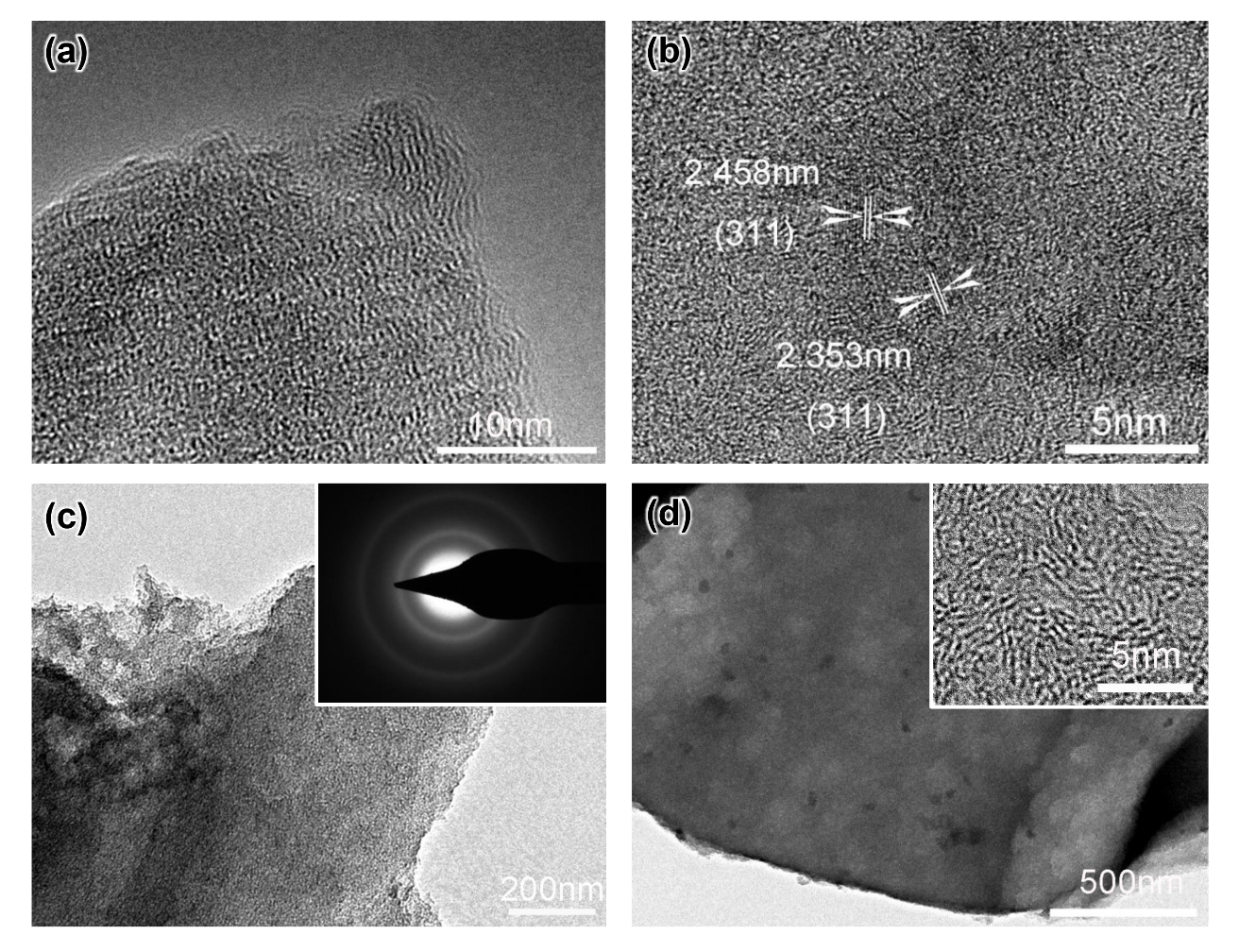
**

**Figure S10** (a, b) TEM images of PHC/Co_3_O_4_; (c-d) TEM images of PHC.

| Co ion concentration in solution | **PHC/Co_3_O_4_** | | **PHC** | | **Standard Sample** | |
| --- | --- | --- | --- | --- | --- | --- |
|  | Int.(c/s) | solution concentration | Int.(c/s) | solution concentration | Int.(c/s) | solution concentration |
| 1^st^ measurement | 119.574 | 1.00239 ppm | 2.43731 | -0.001783 ppm | 6.65874 | 0.00000 ppm  (0.0000 ppm) |
|  | 112.693 |  | 6.11038 |  | 3.64928 |  |
|  | 117.893 |  | 2.49898 |  | 1.34068 |  |
| 2^nd^ measurement | 111.943 | 0.971289 ppm | 3.11675 | -0.004981 ppm | 278.914 | 2.42238 ppm  (2.5000 ppm) |
|  | 113.240 |  | 1.88525 |  | 276.535 |  |
|  | 114.474 |  | 4.96447 |  | 274.245 |  |
| 3^th^ measurement | 117.029 | 1.00906 ppm | 3.24723 | 0.003492 ppm | 570.016 | 5.01673 ppm  (5.0000 ppm) |
|  | 115.554 |  | 6.59801 |  | 565.071 |  |
|  | 119.829 |  | 2.98273 |  | 570.730 |  |
| 4^th^ measurement | 118.811 | 1.00845 ppm | 5.47390 | 0.006550 ppm | 1137.56 | 10.0553 ppm  (10.000 ppm) |
|  | 109.535 |  | 3.62868 |  | 1139.18 |  |
|  | 123.860 |  | 4.75805 |  | 1130.63 |  |
| Co_3_O_4_ content in sample | 18.114 wt% | | 0.011 wt% | | —— | |

**Table S1** The ICP signals of 200 ml HNO_3_ solution (enough to dissolution Co_3_O_4_) immersed 15 mg PHC/Co_3_O_4_ and PHC then take out 20 ml filtered solution attenuation to 200 ml and the Standard samples.

c(Co_3_O_4_)= m/M

m=‾c(Co2^+^) × R × M(Co_3_O_4_)/M(Co_3_)

‾c(Co2^+^)= [c_1_(Co2^+^) + c_2_(Co2^+^) + c_3_(Co2^+^) + c_4_(Co2^+^)]/4

For example, we can calculate the Co_3_O_4_ content in PHC/Co_3_O_4_ as follows:

‾c(Co2^+^)= (1.00239 ppm+0.971289 ppm+1.00906 ppm+1.00845 ppm)/4

= 0.99779725 ppm

m= c(Co2^+^)/4×4000× (59×3+16×4)/(59×3)

= 0.99779725 ppm×2000×241/177

= 2717.1654 μg = 2.717654 mg

c(Co_3_O_4_) = m/M= m/15 mg= 2.717654/15= 18.114%

In this equations, c(Co_3_O_4_) refer to Co_3_O_4_ content in sample; M and m refer to the mass of sample (M=15mg in this experiment) and the mass of Co_3_O_4_ in sample; ‾c (Co2^+^) refer to the average concentration of Co ion in solution; R represent the dilution ratio and it is a fixed value (2000) in this experiment; M(Co_3_O_4_) and M(Co_3_) symbolize the molar mass of Co_3_O_4_ and three molar mass of Co ion. c_1_(Co2^+^), c_2_(Co2^+^), c_3_(Co2^+^) and c_4_(Co2^+^) refer to the concentration of Co ion in solution under different test times.

 **Figure S11** The Co ion intensity signal images of PHC/Co_3_O_4_, PHC, and Standard sample (S. T.) attenuation solutions tested by ICP.

**
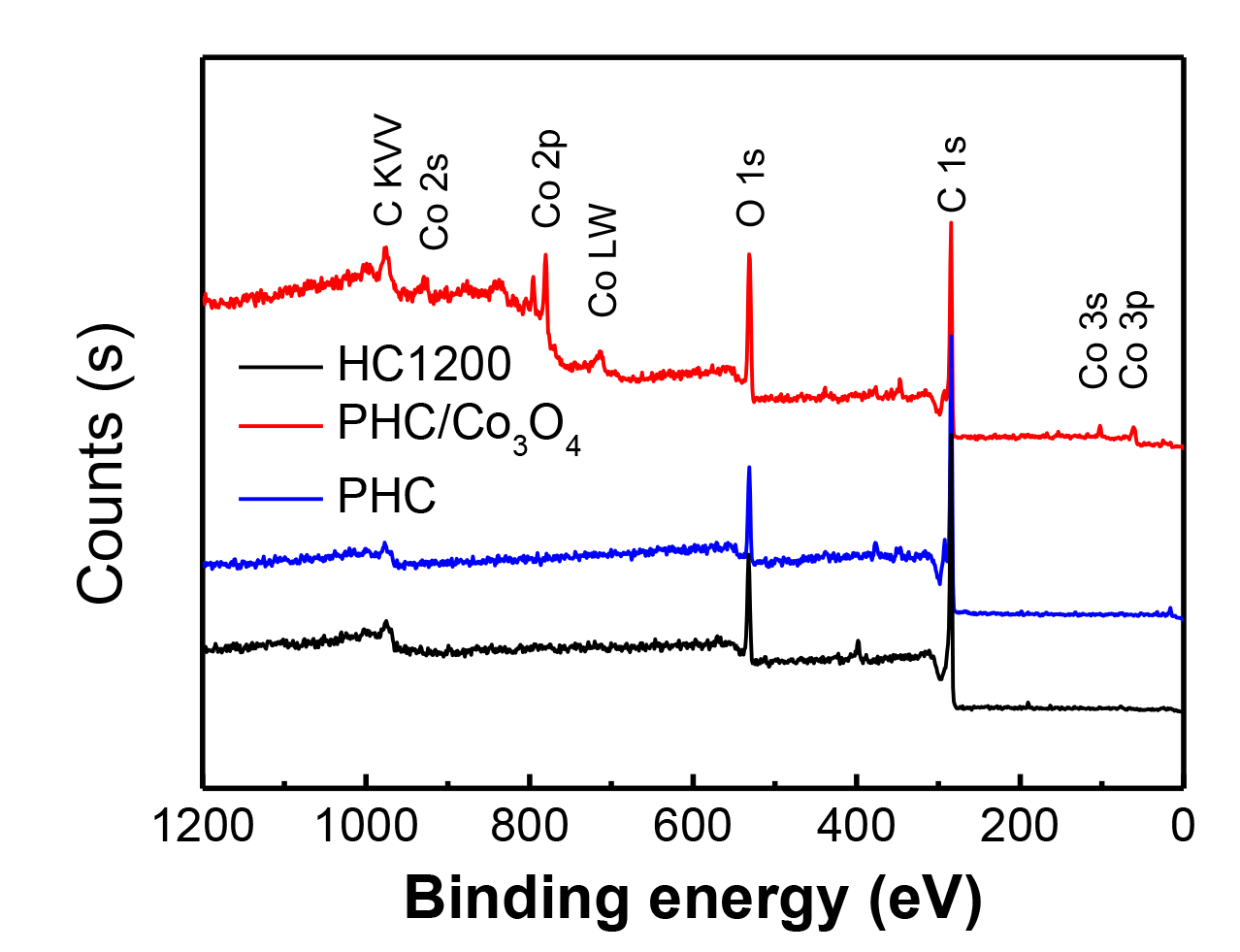
**

**Figure S12** XPS full spectra of HC1200; PHC/Co_3_O_4_ and PHC.

**Figure S13** CV curves of (a) PHC/Co_3_O_4_ and (b) PHC. (c)PHC/ Co_3_O_4_ at low scan rate.

**
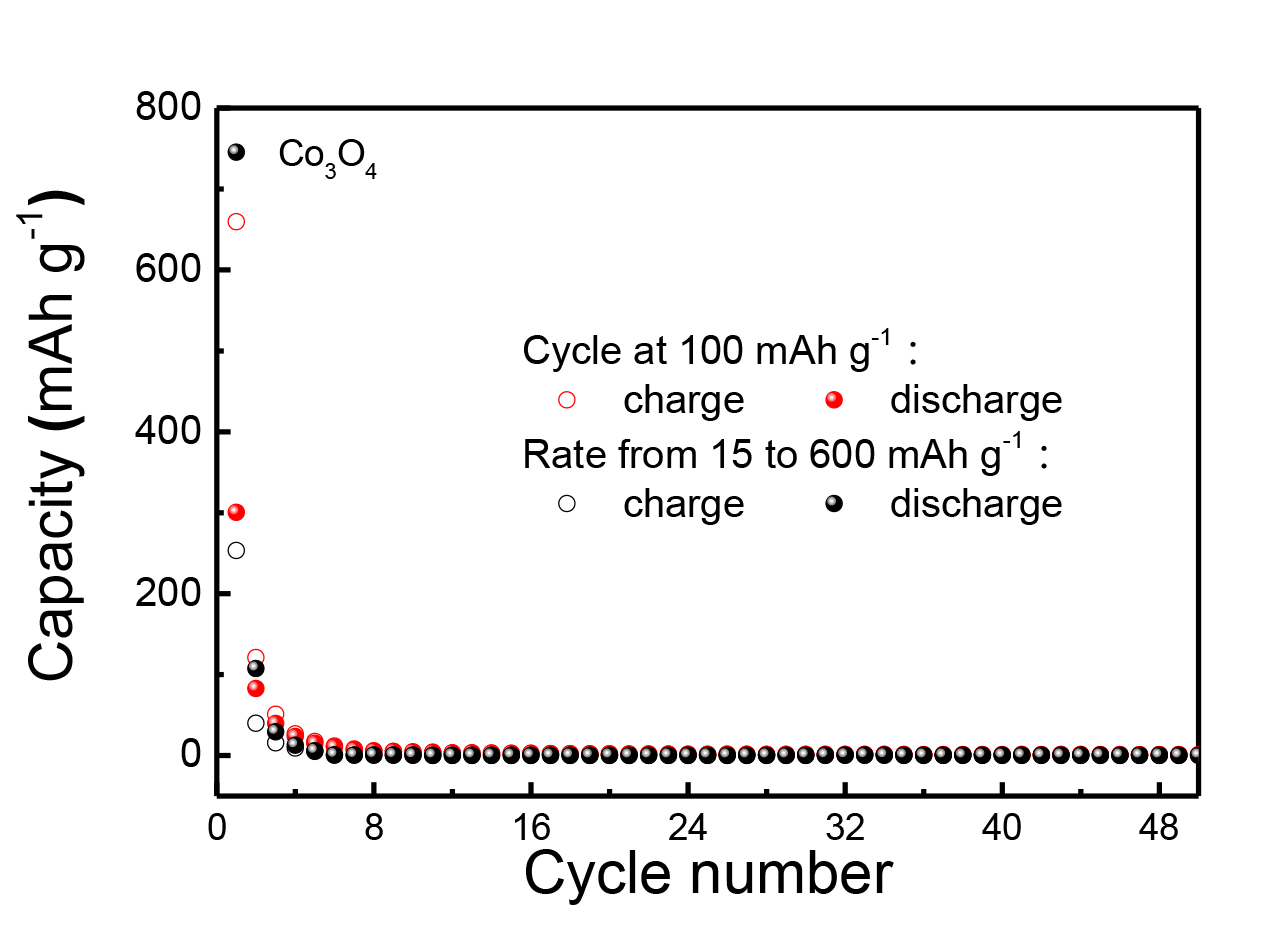
**

**Figure S14** Rate and cycling performances of pure Co_3_O_4_.

**
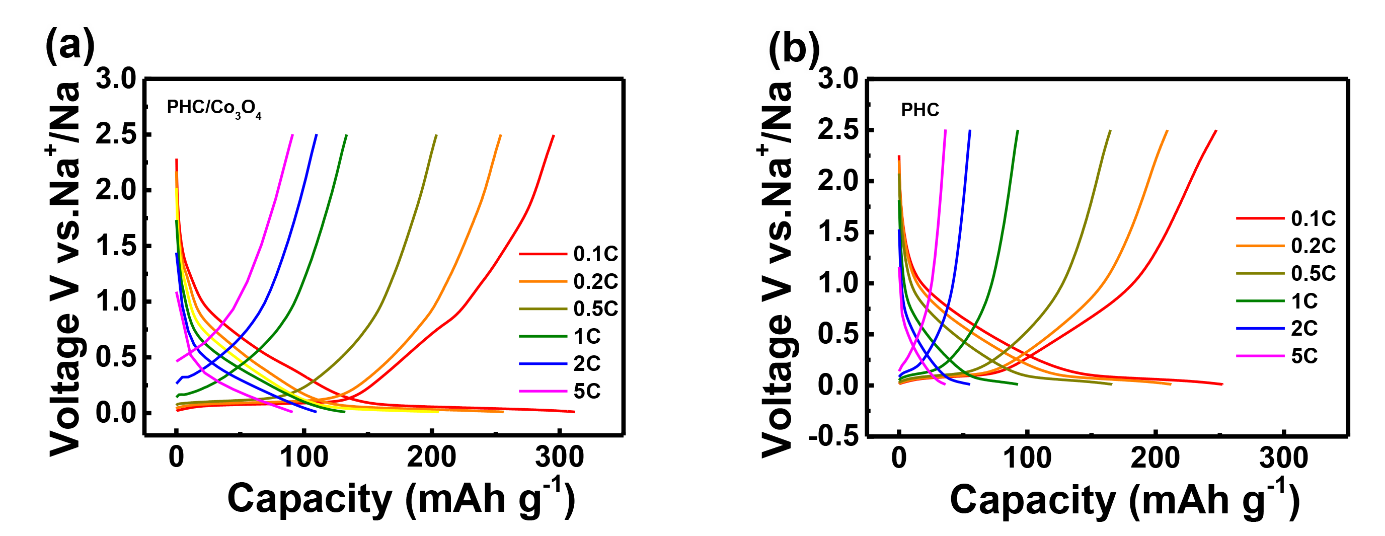
****Figure S15** Charging/ discharging curves of PHC/Co_3_O_4_ (a) and PHC (b) at different rate.

**Figure S16** The charge/discharge curve of full cells.
